# Immunometabolism in Cardiac Remodeling: Mechanisms and Therapeutic Perspectives

**DOI:** 10.3390/ijms27093906

**Published:** 2026-04-28

**Authors:** Julia Nazaruk, Barbara Bilnik, Maciej Niewiadomski, Wojciech Pawlak, Piotr Gajewski

**Affiliations:** 1 Student Scientific Organization, Institute of Heart Diseases, Wroclaw Medical University, 50-556 Wrocław, Poland; julia.nazaruk@student.umw.edu.pl (J.N.); barbara.bilnik@student.umw.edu.pl (B.B.); wojciech.pawlak@student.umw.edu.pl (W.P.); 2 Institute of Heart Diseases, Wroclaw Medical University, 50-556 Wrocław, Poland; dr.piotr.gajewski@gmail.com

**Keywords:** cardiac remodeling, immunometabolism, molecular mechanisms, inflammation, cardiac fibrosis

## Abstract

Cardiovascular diseases remain the leading cause of mortality worldwide, and one of the key mechanisms driving the development of heart failure is pathological remodeling of the myocardium. This process involves complex structural, cellular, and metabolic alterations in which the immune system and its interactions with cardiomyocytes and fibroblasts play a central role. The aim of this work was to present the current state of knowledge on immunometabolism in cardiac remodeling and to discuss its pathophysiological relevance and therapeutic potential. This review focuses on the metabolism of cardiac macrophages, highlighting the differences between the pro-inflammatory (M1) and reparative (M2) phenotypes and their impact on inflammation, fibrosis, and myocardial regeneration. The roles of major metabolic pathways, including glycolysis, oxidative phosphorylation, fatty acid oxidation, and glutaminolysis, are discussed, as well as the importance of the NLRP3 inflammasome and efferocytosis in regulating the inflammatory response. Furthermore, the review briefly incorporates recent insights into neutrophil, T cell, and regulatory T cell (Treg) metabolism and their contributions to inflammation, repair, and fibrotic remodeling. Particular attention is also given to cardiac fibroblasts and their metabolic reprogramming during fibrosis, with emphasis on the pivotal role of transforming growth factor-β (TGF-β) signaling. The review further discusses the role of microRNAs as mediators of intercellular communication integrating immunological and metabolic signals. The work is complemented by a discussion of therapeutic perspectives, including modulation of macrophage metabolism, fibrogenic signaling pathways, mitochondrial function, and miRNA-based therapies. Immunometabolism emerges as a promising research field whose further exploration may contribute to the development of novel, more precise strategies for the treatment of cardiovascular diseases.

## 1. Introduction

Cardiovascular diseases are the leading cause of death worldwide. In 2022 alone, 19.8 million deaths were attributed to cardiovascular causes, accounting for approximately 32% of all deaths [1]. Many of these conditions are associated with cardiac dysfunction resulting from remodeling. This term refers to molecular, cellular, and interstitial changes, induced by injury, that may affect the size and function of the myocardium [2,3]. The immune system plays a crucial role in this process. Dying cells produce danger-associated molecular patterns (DAMPs), which recruit macrophages and neutrophils that produce cytokines and reactive oxygen species [4]. The human heart has relatively limited regenerative capacity, mediated by anti-inflammatory macrophage phenotypes, neutrophils, and B and T lymphocytes [5,6]. Immunometabolism is defined as changes in the metabolism of immune cells that influence their functions [7].

Physiological remodeling takes place during pregnancy and as a result of physical exercise. In both cases, it is adaptive in nature, and cardiac function remains normal [8,9,10]. Pathological remodeling is a consequence of activation of the Gαq signaling pathway, whereas under physiological conditions, the PI3K pathway predominates [8,11]. This leads to a cascade resulting in irreversible myocardial hypertrophy, characterized by fibrosis, impaired angiogenesis, and deteriorated cardiac function. Various factors inducing metabolic stress in cardiomyocytes may contribute to this process, including myocardial infarction, hypertension, obesity, and cardiac arrhythmias, including those induced by pacemaker stimulation [12,13,14]. Studies also indicate an association between epicardial adipose tissue and the function of the left atrium [15].

The aim of this review is to present current knowledge on immunometabolism in the context of cardiac remodeling. The review covers metabolic mechanisms of macrophages, neutrophils and lymphocytes, the role of fibroblasts in fibrosis and the importance of miRNAs. In addition, potential therapeutic perspectives based on the modulation of metabolic pathways are discussed.

## 2. Immunometabolism of Macrophages in the Heart

### 2.1. M1 and M2 Macrophages: Characterization and Role After Cardiac Injury

Contrary to popular belief, cardiomyocytes do not constitute the majority of cardiac cells, accounting for only approximately 30%. The remaining cells include immune cells, smooth muscle cells, fibroblasts, pericytes, and many others that are essential for proper cardiomyocyte function. Cardiac macrophages constitute up to 6–10% of all cardiac cells [16]. Although single-cell RNA sequencing (scRNA-seq) studies have revealed substantial heterogeneity of macrophages in the human body, the classification into classically activated M1 macrophages and alternatively activated M2 macrophages remains useful [17,18,19]. The M1 phenotype is activated by danger-associated signals such as bacterial lipopolysaccharide (LPS) or interferon gamma (IFN-γ), as well as signals related to cellular damage, including pathogen-associated molecular patterns (PAMPs) and damage-associated molecular patterns (DAMPs) [20,21]. M1 macrophages are characterized by high expression of major histocompatibility complex (MHC) class II, cluster of differentiation 80 (CD80), CD68, and inducible nitric oxide synthase (iNOS) [20]. They phagocytose debris from parenchymal and stromal cells, degrade the extracellular matrix (ECM), and promote further propagation of inflammation through the production of tumor necrosis factor α (TNF-α), interleukin-1 beta (IL-1β), and IL-6. Clearance of dying cells may limit inflammation by preventing secondary necrosis of surrounding tissues [17,22]. Studies also demonstrate that M1 macrophages inhibit angiogenesis and exacerbate cardiac injury through the secretion of microRNAs such as miR-155 [23]. It should be noted that macrophage polarization is dynamic and depends more on the current microenvironment than on previous activation states [24]. The M2 macrophage phenotype is activated by cytokines produced by T helper 2 (Th2) lymphocytes. Four subclasses are distinguished: M2a, M2b, M2c, and M2d. These macrophages generally exhibit anti-inflammatory properties, with the exception of M2b, which may also display pro-inflammatory features through IL-1β production [20]. Their anti-inflammatory function is mediated by the production of cytokines such as IL-10 and TGF-β, along with reduced expression of pro-inflammatory IL-12 and IL-23 [25]. Excessive stimulation of M2 macrophages may lead to tissue fibrosis [26].

### 2.2. Key Metabolic Pathways of Macrophages

Distinct metabolic profiles of M1 and M2 macrophages are crucial for their respective functions. Metabolism is not only a consequence but also a driver of macrophage polarization [27]. 

M1 macrophages primarily generate energy through glycolysis, supported by increased expression of the glucose transporter GLUT-1. Enhanced glucose uptake leads to increased production of mitochondrial reactive oxygen species (mROS), which promotes dimerization of pyruvate kinase M2 (PKM2). PKM2 subsequently translocates to the nucleus, where it phosphorylates the transcription factor STAT3, thereby increasing the production of IL-1β and IL-6 [22,28].

The tricarboxylic acid (TCA) cycle in M1 macrophages does not proceed in a canonical manner. Reduced expression of succinate dehydrogenase and isocitrate dehydrogenase results in the accumulation of succinate and citrate. Elevated citrate levels promote phospholipid synthesis required for membrane formation and may also influence IL-1β production [29]. Succinate stabilizes hypoxia-inducible factor 1-alpha (HIF-1α), which is also stabilized under hypoxic conditions and promotes transcription of glycolytic enzymes as well as IL-1β [30]. Glycolysis has also been shown to play a key role in inflammatory monocyte adhesion [31]. 

The pentose phosphate pathway (PPP) is upregulated in M1 macrophages, enabling production of nicotinamide adenine dinucleotide phosphate (NADPH), which is involved in fatty acid synthesis and generation of inflammatory mediators, as well as pentose phosphates required for amino acid and nucleotide metabolism. PPP activity is enhanced in the presence of LPS due to reduced expression of the inhibitory carbohydrate kinase-like protein (CARKL). In contrast, M2 macrophages exhibit increased CARKL expression, leading to suppression of the PPP [20,32]. 

M2 macrophages rely on oxidative phosphorylation (OXPHOS) and fatty acid oxidation (FAO) for energy production. In this phenotype, the TCA cycle remains intact. Fatty acids are taken up from lipoproteins via CD36. FAO plays a key role in M2 polarization; inhibition of FAO with etomoxir has been shown to impair monocyte and macrophage polarization toward the M2 phenotype [33,34]. 

**Figure 1:**
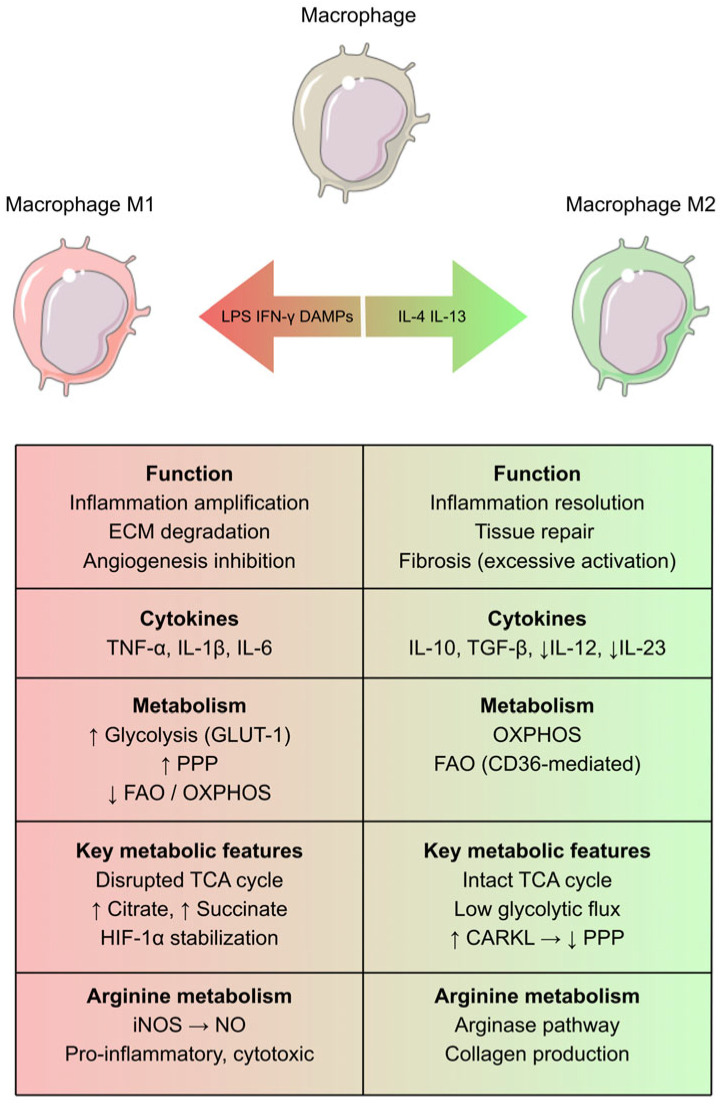
Functional and metabolic differences between M1 and M2 macrophages.

M1 and M2 macrophages also differ in arginine metabolism. M1 macrophages utilize arginine via iNOS to produce citrulline and nitric oxide (NO). Nitric oxide promotes inflammation and, in excessive amounts, may damage cardiomyocytes. In contrast, M2 macrophages metabolize arginine through arginase, a pathway that enhances cell proliferation and stimulates collagen fiber production [20,35]. A simplified version of the above-mentioned processes is presented in Figure 1.

### 2.3. Cytokines: IL-1β, IL-6, TNF-α—Their Role in Inflammation and Regeneration

IL-1β is a pro-inflammatory cytokine that induces the production of numerous other inflammatory mediators. It is synthesized as an inactive precursor (proIL-1β) and requires activation by the inflammasome complex, where caspase-1 cleaves it into its active form [36]. IL-1β expression is associated with activation of the NF-κB pathway, which is triggered by stimulation of Toll-like receptor 4 (TLR4) by LPS or by components released from dying cells [37]. This pathway operates in many cell types, including macrophages, and promotes M1 polarization while acting as a transcription factor driving cytokine and chemokine production [38]. IL-1β signals through the type 1 IL-1 receptor (IL-1R1), initiating an inflammatory cascade that recruits pro-inflammatory leukocytes and stimulates ECM degradation programs in fibroblasts, while delaying their differentiation into myofibroblasts. It also induces matrix metalloproteinase production and promotes fibroblast migration, thereby contributing to cardiac fibrosis [37,39]. IL-1β and other NF-κB-dependent products can also damage the endothelium, including that of the aortic valve, leading to calcified aortic valve disease and ultimately heart failure [37]. Notably, the CANTOS trial demonstrated a 15% reduction in cardiovascular events following administration of canakinumab, an antibody blocking IL-1β, compared with placebo [40].

Another important finding from the CANTOS trial was that patients with a reduction in IL-6 levels greater than the median experienced a 36% reduction in cardiovascular events. IL-6, like IL-1β, is produced by M1 macrophages. It signals through three pathways, but in most cells it acts via trans-signaling involving the soluble IL-6 receptor (sIL-6R), which binds IL-6 extracellularly and subsequently associates with gp130 on target cells [41]. While early inflammation is beneficial for the clearance of dead cells and the initiation of repair processes, chronically elevated IL-6 levels are associated with increased risk of cardiovascular mortality, including myocardial infarction and heart failure. IL-6 also damages vascular endothelium, promoting atherosclerosis [42]. IL-6 contributes to cardiac fibrosis by enhancing fibroblast proliferation [37].

Another pro-inflammatory cytokine secreted by M1 macrophages is TNF-α, which can promote remodeling through induction of fibrosis [43]. Mouse studies indicate that elevated TNF-α levels stimulate production of matrix metalloproteinases that degrade the extracellular matrix. Long-term degradation of collagen fibers ultimately leads to ventricular dysfunction. TNF-α also contributes to cardiomyocyte apoptosis [44].

### 2.4. NLRP3 Inflammasome and Calprotectin

The nucleotide-binding oligomerization domain-like receptor family pyrin domain-containing 3 (NLRP3) inflammasome is a multiprotein complex responsible for activation of IL-1β and IL-18. It consists of three components: NLRP3, apoptosis-associated speck-like protein (ASC), and caspase-1, which cleaves proIL-1β into its active form. Activation of the inflammasome occurs in two stages: priming and activation. Priming involves synthesis of inflammasome components through activation of the NF-κB transcription factor, sometimes also driven by IL-1β and TNF. Final activation is triggered by DAMPs acting on TLRs [45]. Intense glycolysis in M1 macrophages is linked to NLRP3 activation. Glycolytic enzymes such as hexokinase 1 and pyruvate kinase M2 enhance its activation. Lactate, a product of glycolysis, also promotes NLRP3 activation. Disruption of the TCA cycle in M1 macrophages further contributes to this process, as citrate accumulation increases expression of NLRP3 and proIL-1β [30]. Excessive IL-1β production by the activated inflammasome in M1 macrophages indirectly promotes cardiac remodeling through fibrosis and endothelial dysfunction, as discussed earlier. Moreover, studies in rats have demonstrated that advanced heart failure may be associated with increased NLRP3 expression in the lungs as well as with renal fibrosis [46].

An important factor influencing, among others, the NLRP3 inflammasome is the heterodimeric complex S100A8/A9, also known as calprotectin. It is predominantly produced by monocytes and neutrophils and exerts its effects through Toll-like receptor 4 (TLR4) and the receptor for advanced glycation end products (RAGE). In addition, it plays a role in the recruitment of immune cells. Studies have demonstrated an association between elevated calprotectin levels, for example following myocardial infarction, and enhanced inflammation leading to structural remodeling, left ventricular dysfunction, and arrhythmias. Furthermore, studies in experimentally induced myocarditis in mice have shown that short-term administration (3–5 or 7 days) of the S100A9 inhibitor ABR-238901 improved survival and cardiac function [47].

### 2.5. Intercellular Interactions and Their Impact on Cardiomyocytes

Efferocytosis is the process by which apoptotic cells are engulfed by phagocytes, including macrophages. Uptake of apoptotic cells delivers large amounts of nutrients such as lipids, nucleic acids, and amino acids. Macrophages must undergo metabolic reprogramming; otherwise, resolution of inflammation does not occur. The binding of apoptotic cells increases glucose uptake via the transporter solute carrier family 2 member 1 (SLC2A1). Glycolysis is required for actin polymerization, necessary for continued efferocytosis. Lactate is also produced and exported through the SLC16A1 transporter, where it acts as a paracrine signal stimulating neighboring cells to produce anti-inflammatory IL-10 and TGF-β. Excess DNA is degraded by lysosomal DNase 2. Efferocytosis promotes macrophage polarization toward the M2 phenotype, characterized by OXPHOS and FAO, leading to increased production of anti-inflammatory interleukins [48]. A study published in 2024 demonstrated that impaired efferocytosis exacerbated cardiac dysfunction by increasing cardiomyocyte death, inflammation, and fibrosis [49].

Cardiomyocytes can also transfer damaged mitochondria and other components via vesicles known as exophers. Macrophages can engulf and degrade these structures through the phagocytic receptor Mer tyrosine kinase (MerTK). Accumulation of dysfunctional mitochondria in cardiomyocytes has been shown to disrupt their metabolism and lead to ventricular dysfunction. A reciprocal mechanism may also occur, whereby cardiomyocytes take up mitochondria from macrophages via clathrin-mediated endocytosis. This process induces ferroptosis, an iron-dependent form of cell death characterized by phospholipid peroxidation [50,51].

## 3. Neutrophils and Lymphocytes in Cardiac Immunometabolism

### 3.1. Neutrophils

Neutrophils are among the earliest immune cells recruited to the injured myocardium and play a central role in initiating the acute inflammatory response that precedes cardiac remodeling. Within hours after tissue injury, they accumulate in the affected area and release a broad range of mediators, including IL-1β, TNF-α, chemokines, reactive oxygen species, and matrix metalloproteinases (MMPs), which contribute to the clearance of necrotic debris but may also exacerbate local tissue damage [52]. Their effector functions, such as reactive oxygen species production and the formation of neutrophil extracellular traps (NETs), are essential for host defense. However, in sterile inflammatory conditions, including ischemic injury, these mechanisms can amplify oxidative stress, promote endothelial dysfunction, and accelerate maladaptive remodeling [53,54]. In addition, neutrophils can undergo NETosis, a specialized form of cell death leading to the release of NETs, which, although initially protective, have been implicated in sustaining sterile inflammation and promoting adverse cardiac remodeling through mechanisms involving myeloperoxidase (MPO), neutrophil elastase (NE), and peptidylarginine deiminase 4 (PAD4) [55].

Neutrophils also actively participate in extracellular matrix turnover and fibrogenic signaling. In addition to releasing proteolytic enzymes that degrade matrix components, they have been shown to induce TGF-β expression in proliferating fibroblasts, thereby linking early inflammation with subsequent fibrotic remodeling [53]. Importantly, neutrophil-driven processes are closely associated with metabolic adaptation. Under hypoxic conditions characteristic of injured myocardium, neutrophils undergo a shift toward glycolysis, driven in part by hypoxia-inducible factors (HIFs) and pattern recognition receptor signaling. This metabolic reprogramming supports rapid ATP generation and sustains key inflammatory functions, including reactive oxygen species production, which is further amplified through the pentose phosphate pathway via NADPH generation [54,56].

Despite their well-established pro-inflammatory role, neutrophils also contribute to the resolution of inflammation and tissue repair. Their functional heterogeneity has been highlighted by the identification of distinct phenotypes, with pro-inflammatory subsets promoting adverse remodeling and anti-inflammatory populations exerting protective effects [54]. Moreover, neutrophils influence macrophage behavior by releasing mediators that promote a reparative phenotype and enhance clearance of cellular debris. During the resolution phase, apoptotic neutrophils expose signals that facilitate their recognition and removal by macrophages, supporting the transition toward tissue repair [52]. Notably, experimental depletion of neutrophils has been associated with increased fibrosis and impaired cardiac function, underscoring their context-dependent role in maintaining balanced remodeling [54].

Taken together, neutrophils represent a highly dynamic and metabolically adaptable component of the immune response, whose tightly regulated activity determines whether early inflammation resolves efficiently or progresses toward chronic tissue damage and fibrosis.

### 3.2. T Lymphocytes

The metabolic state of T cells infiltrating cardiac tissue regulates the progression from repair to the process of fibrosis. This localized cardiac response follows the metabolic principle where quiescent T cells primarily rely on OXPHOS for ATP production, while activated T cells typically shift away from lipid oxidation in favor of glucose consumption via aerobic glycolysis [57]. This metabolic activity can change under physiological stress, which weakens the cells’ cytotoxic and effector function [58]. The identified cause of this phenomenon is overproduction of ROS, the specific mitochondrial effect which will be explained in a section covering oxidative stress.

Through metabolic shifts, these lymphocytes drive structural changes and the buildup of extracellular matrix. Effector T cells can function as key players in the structural changes that occur in heart tissue during cardiovascular disease. In this context, aggressive effector subsets such as cytotoxic CD8+ cells and proinflammatory CD4+ cells such as Th1 and Th17 drive fibrotic progression by stimulating fibroblast proliferation and the expansion of matrix [59]. Mirroring the behavior of M1 macrophages, Th17 lymphocytes in the damaged heart undergo metabolic reprogramming toward aerobic glycolysis, that is driven by HIF-1α activation and increased GLUT1 expression, which facilitates rapid glucose intake, allowing for prolific secretion of proinflammatory cytokines like IL-17 and TNF-α over the more efficient oxidative phosphorylation used in homeostatic conditions [27,57].

These metabolic and immunological responses are tightly coordinated by CD4+ helper T cells, which respond to signals from the microenvironment. A key component of communication is the cell membrane, whose function relies on the specific composition of membrane lipids, namely cholesterol, which stabilizes lipid rafts and prevents spontaneous activation of the T-cell receptor (TCR), and glycosphingolipids that interact with the TCR to influence lymphocyte differentiation [60]. This is particularly evident in the shift toward Th17 cells, which are critical in inflammatory as well as autoimmunity processes [60]. Consequently, the lipid composition of membranes is a key factor in how accurately these immune cells respond to their environment.

Internally, T cell activation depends heavily on the availability of cholesterol. In CD8+ cells, this is primarily supplied through the Low-Density Lipoprotein receptor (LDLR) pathway, which correlates with higher mRNA and protein expression of LDLR in activated CD8+ cells than CD4+ cells [60]. When this pathway is disrupted, it weakens mTORC1 signaling, leading to mush lower proliferation and cytokine production, including IFN-gamma, Granzyme B and perforin [60]. Furthermore, the LXR receptor serves as a major controller of lipid metabolism in human CD4+ lymphocytes, by turning on genes responsible for moving cholesterol (ABCA1, ABCG1) and making fatty acids (SCD, SREBF1) [61]. Presented regulation of T cells demonstrates that cholesterol is much more than just a structural component; it acts as a metabolic gatekeeper that controls the cells’ proliferation and cytotoxic activity. In the heart, an excess of cholesterol in the local environment can directly drive inflammation, eventually leading to permanent damage to the heart’s structure via fibrosis.

### 3.3. Regulatory T Lymphocytes

Regulatory T lymphocytes constitute a specialized subset of CD4+ T cells that play a central role in maintaining immune homeostasis and restraining excessive inflammation during cardiac remodeling. These cells are defined by the expression of the transcription factor FOXP3, which governs their suppressive phenotype and functional stability [62]. Following myocardial injury, Treg cells actively contribute to the resolution of inflammation by limiting the activity of pro-inflammatory immune populations, including M1 macrophages, neutrophils, and effector T cells, while at the same time supporting reparative processes such as angiogenesis and tissue regeneration [63,64].

The functional capacity of Treg cells is closely linked to their metabolic state. In contrast to effector T cells, which predominantly depend on glycolysis, Treg cells rely primarily on oxidative phosphorylation and fatty acid oxidation, although increasing evidence highlights their metabolic flexibility depending on tissue context and activation state [65,66]. This metabolic phenotype is tightly controlled by key signaling pathways, including AMP-activated protein kinase (AMPK) and the mammalian target of rapamycin (mTOR), which integrate environmental cues to regulate Treg differentiation and stability [66,67]. In particular, AMPK activation supports oxidative metabolism and Treg suppressive function, whereas excessive mTOR signaling may impair Treg stability and promote loss of their regulatory phenotype. Disruption of these pathways has been associated with impaired immunoregulatory function and may contribute to sustained inflammation and fibrotic remodeling.

Importantly, Treg cells exhibit substantial phenotypic and functional heterogeneity and can differentiate into tissue-adapted populations with organ-specific transcriptional programs [68]. Distinct subsets have been identified based on activation status and tissue localization, including naive, effector, and tissue-resident Treg populations. In the cardiac environment, tissue-resident Treg cells produce anti-inflammatory mediators such as IL-10 and TGF-β, as well as growth factors that directly modulate fibroblast activity and extracellular matrix remodeling [63,69]. Collectively, these findings position Treg cells as key immunometabolic regulators that integrate immune suppression with metabolic adaptation, thereby influencing the balance between inflammation, repair, and fibrosis in the remodeling myocardium.

## 4. Fibroblasts and Cardiac Fibrosis

Cardiac fibrosis appears to be a central pathological process in most forms of heart failure [70]. It is generally described as the excessive deposition of ECM components, primarily collagen, within the myocardial interstitium [43]. Interestingly, fibrosis may serve a dual role: in the acute phase, it may help reinforce the structural integrity of the cardiac wall, potentially preventing rupture, whereas chronic persistence is associated with increased myocardial stiffness, impaired electrical conduction, and progressive deterioration of systolic and diastolic function [71]. Fibroblasts are generally considered the principal cells responsible for fibrosis; when exposed to injury-related stimuli, they become activated and differentiate into myofibroblasts. Traditionally, mechanical overload and neurohormonal activation have been viewed as the main drivers of cardiac fibrosis. However, accumulating evidence indicates that immune modulation, chronic inflammation, and metabolic disturbances also play critical roles in its initiation and progression [54,71]. Within this framework, metabolic reprogramming of fibroblasts has emerged as a key mechanism regulating their activation, phenotypic plasticity, and contribution to structural remodeling of the heart. Importantly, these processes are increasingly understood in the context of immunometabolism, whereby coordinated metabolic changes in both immune cells and fibroblasts shape the inflammatory microenvironment and drive structural remodeling of the heart [54,56,72].

### 4.1. Transformation of Fibroblasts into Myofibroblasts

The shift from quiescent fibroblasts to activated, synthetically and contractile myofibroblasts is widely considered central to fibrosis development [71]. While resident fibroblasts are the main contributors, evidence indicates that other cell types, such as macrophages, endothelial cells, and pericytes, may also contribute to the myofibroblast population [71,73]. Some recent studies even suggest that macrophages could directly transdifferentiate into myofibroblasts, though the extent of this contribution remains under investigation [74].

Cardiac fibroblasts, as mesenchymal-derived interstitial cells, play a critical role in maintaining ECM integrity. Under normal conditions, they manage ECM turnover through carefully regulated synthesis and degradation, showing relatively low metabolic activity and limited collagen production. Importantly, these cells are heterogeneous and retain a capacity for dynamic phenotypic adaptation in response to injury, though the exact triggers and mechanisms remain incompletely understood [71].

Upon activation, fibroblasts acquire a myofibroblast phenotype characterized by the expression of α-smooth muscle actin (α-SMA), enhanced capacity for ECM synthesis and organization, and active participation in matrix remodeling through the MMPs and their inhibitors (TIMPs) [71,73]. Although the mechanisms of myofibroblast activation depend on the type of injury triggering cardiac fibrosis, several common pathways can be identified [71]. Fibroblasts can be directly stimulated by fibrogenic mediators or indirectly through inflammatory and paracrine signals from immune cells, vascular cells, or cardiomyocytes. Cytokines and growth factors, including TGF-β, along with ECM proteins secreted by macrophages, lymphocytes, mast cells, and eosinophils, appear to enhance fibroblast activation. Cardiomyocytes under mechanical or metabolic stress may also release fibrogenic signals [71]. Additionally, changes in ECM stiffness due to collagen accumulation are likely important in promoting fibroblast activation [75,76].

Fibroblast activation and the acquisition of a myofibroblast phenotype are associated with increased energetic demands and profound metabolic reprogramming, enabling intensive ECM synthesis. Importantly, these processes are increasingly recognized as being governed by immunometabolic signals, whereby inflammatory mediators modulate fibroblast metabolism, which in turn shapes their activation and phenotypic plasticity [54,77].

### 4.2. The Role of Metabolism in the Fibrotic Process

Cardiac fibrosis is an active and highly regulated process rather than a passive accumulation of extracellular matrix components. Increasing evidence indicates that metabolic reprogramming is a key factor enabling the transformation of fibroblasts into myofibroblasts and sustaining their profibrotic activity. In this context, cellular metabolism not only supplies energy but also functions as an integral regulator of cell phenotype, gene expression, and interactions with the inflammatory microenvironment, fitting within the broader concept of immunometabolism [54,72,77]. Recent transcriptomic and proteomic analyses in arrhythmogenic cardiomyopathy have identified dysregulated metabolic and immune pathways associated with fibroblast activation and fibrotic remodeling, highlighting the importance of immunometabolic regulation in cardiac fibrosis [78].

Under physiological conditions, cardiac fibroblasts exhibit relatively low metabolic activity and preferentially rely on FAO coupled with OXPHOS. This metabolic profile supports the maintenance of a quiescent phenotype and ensures stable ATP production required for basal ECM turnover [27,54,72]. Cardiac fibrosis, however, represents a highly anabolic process that demands substantial energy input and a continuous supply of biosynthetic precursors. Collagen synthesis involves energy-intensive protein translation, post-translational amino acid modifications, proper folding of the triple helix, and active secretion of ECM components. The increased demand for ATP, amino acids, and metabolic intermediates necessitates supplementation of oxidative pathways such as FAO and OXPHOS with anabolic metabolic routes that provide precursors for intensive collagen synthesis [27].

In response to these demands, fibroblasts undergo metabolic reprogramming characterized by a shift from OXPHOS dominance toward enhanced glycolysis, even under normoxic conditions. Glycolysis enables rapid ATP generation and supplies intermediates required for the biosynthesis of nucleotides, lipids, and amino acids. Concurrent suppression of FAO facilitates the loss of the quiescent phenotype and promotes adoption of the myofibroblast program [54]. An additional key component of fibroblast metabolic adaptation is enhanced glutaminolysis. In models of myofibroblast differentiation, increased glutaminolysis elevates the availability of α-ketoglutarate (αKG), which not only supports metabolic requirements but also influences epigenetic regulation of profibrotic gene expression, thereby stabilizing the myofibroblast phenotype. Inhibition of glutaminase suppresses α-SMA expression and collagen synthesis, whereas αKG supplementation restores myofibroblast characteristics, underscoring the central role of this metabolic pathway in fibroblast activation [79].

Metabolites generated during fibroblast metabolic reprogramming act as signaling mediators that integrate metabolic and inflammatory pathways. For instance, succinate stabilizes HIF-1α and promotes IL-1β production, thereby linking cellular metabolism with pro-inflammatory signaling. Moreover, lactate, a product of increased glycolysis, stimulates fibroblast activation and supports their differentiation into myofibroblasts. Such metabolic–inflammatory crosstalk may indirectly support profibrotic fibroblast activation within the injured myocardium [54,56,80].

In addition to classical metabolic intermediates, DAMPs such as the S100A8/A9 complex have emerged as important links between inflammation and fibrotic remodeling. Released by activated neutrophils and monocytes/macrophages in response to tissue injury, S100A8/A9 amplifies inflammatory signaling through receptors such as TLR4 and RAGE, promoting NF-κB-dependent cytokine production. This pro-inflammatory environment may indirectly enhance fibroblast activation and support their metabolic reprogramming toward a profibrotic phenotype. Moreover, S100A8/A9 has been associated with glycolytic shifts in immune cells, further sustaining inflammation and creating conditions that favor persistent myofibroblast activation and extracellular matrix deposition [47].

### 4.3. The TGF-β Pathway as a Central Regulator of Fibrogenesis

TGF-β is one of the principal regulators of fibrogenesis and fibroblast activation in the injured myocardium. Its actions include induction of fibroblast-to-myofibroblast differentiation, stimulation of extracellular matrix synthesis, and stabilization of the ECM through the induction of TIMPs and plasminogen activator inhibitor-1 (PAI-1). Experimental studies have demonstrated that TGF-β signaling is indispensable for the development of cardiac fibrosis [71].

In the heart, TGF-β is predominantly present in a latent form bound to the extracellular matrix, and its local activation occurs in response to tissue injury through the action of proteases, integrins, and changes in the biomechanical properties of the ECM. In addition, TGF-β is produced by multiple cell types, including macrophages, fibroblasts, cardiomyocytes, and platelets, highlighting its role as a key integrator of inflammatory responses and tissue remodeling [54,71].

TGF-β signaling is transmitted intracellularly via the canonical Smad-dependent pathway. Upon ligand binding, the type II receptor (TGF-βR2) activates the type I receptor (TGF-βR1), which subsequently phosphorylates Smad2 and Smad3. Activated Smads form a complex with Smad4 and translocate to the nucleus, where they regulate the expression of genes involved in fibrogenesis, including those encoding collagen and cytoskeletal proteins characteristic of myofibroblasts. The critical role of this pathway has been confirmed by studies demonstrating that fibroblast-specific activation of TGF-β-Smad2/3 signaling is required for the development of cardiac fibrosis in animal models. Negative regulation of this pathway is mediated by Smad6 and Smad7, which limit receptor activity and promote receptor degradation [81,82].

In addition to canonical Smad2/3 signaling, TGF-β also activates non-canonical pathways, including mitogen-activated protein kinase (MAPK) cascades (ERK1/2, p38, JNK), phosphoinositide 3-kinase (PI3K)/AKT signaling, and the Rho pathway. These mechanisms support the profibrotic response of fibroblasts by modulating their proliferation, survival, and metabolic activity. By integrating growth factor and metabolic signals, non-canonical TGF-β pathways contribute to enhanced ECM synthesis and stabilization of the myofibroblast phenotype, underscoring the complexity of fibrogenic regulation in the heart [81].

In summary, the TGF-β pathway serves as a central regulator of cardiac fibrogenesis, integrating inflammatory, mechanical, and metabolic signals. Its multilevel effects on fibroblasts make it a critical component of cardiac remodeling and an attractive target for potential therapeutic interventions aimed at limiting pathological fibrosis.

### 4.4. ECM Deposition and Formation of Collagen Networks

The cardiac extracellular matrix is a dynamic structure that provides mechanical support for cardiomyocytes and participates in the transmission of biomechanical signals. Activated fibroblasts in the form of myofibroblasts are responsible for the intensive synthesis and organization of ECM components, including type I and III collagen, fibronectin, elastin, and proteoglycans. Type I collagen forms thick, rigid fibers, whereas type III collagen confers tissue elasticity. Alterations in the type I/type III collagen ratio lead to increased ventricular wall stiffness and impaired diastolic function [71,83].

**Figure 2:**
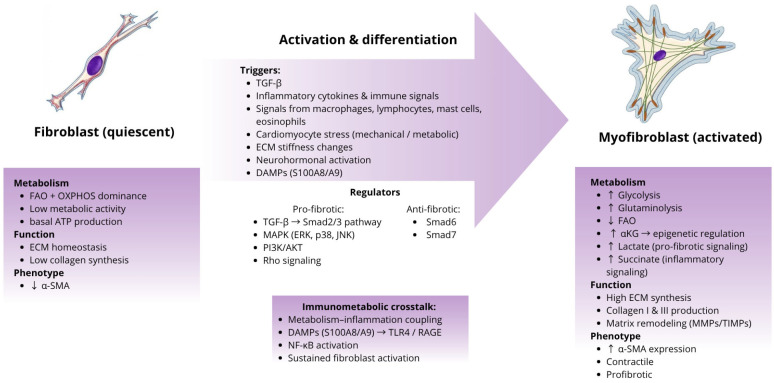
Fibroblast-to-myofibroblast differentiation in fibrotic myocardium. Adapted from Fink et al., CC BY 4.0 https://commons.wikimedia.org/wiki/File:Fibroblast%E2%80%93myofibroblast_differentiation.png (accessed on 23 March 2026).

Excessive accumulation of extracellular matrix in the myocardium disrupts both the mechanical and electrical properties of the heart. Fibrosis increases myocardial stiffness and impairs diastolic function by limiting elastic recoil, which is particularly relevant in the pathogenesis of heart failure with preserved ejection fraction. Moreover, excess collagen interferes with electrical impulse conduction and mechanical coupling between cardiomyocytes, promoting arrhythmogenesis and inefficient coordination of contraction [84]. These processes are a direct consequence of sustained myofibroblast activation and their metabolic reprogramming, which supports long-term synthesis and stabilization of collagen networks within the myocardium [54,71]. This process is presented in Figure 2.

## 5. MicroRNAs

MicroRNAs (miRNAs) represent a crucial component of post-transcriptional gene expression regulatory mechanisms and constitute a key interface integrating metabolic and immunological signaling. These are short, non-coding RNA molecules whose biological activity is based on interactions with specific mRNA transcripts, resulting in reduced translational efficiency or destabilization of target RNA molecules [85]. Through this mechanism, miRNAs enable precise fine-tuning of protein expression in accordance with the current cellular demands, which is particularly relevant in tissues characterized by high metabolic and immunological plasticity, such as the myocardium undergoing remodeling processes.

Within the framework of immunometabolism, miRNAs function as regulators of complex signaling networks that govern both immune cell activity and their metabolic phenotype [7]. They are involved in modulating immune cell proliferation, activation, and differentiation, while concurrently influencing metabolic pathways associated with glucose and lipid utilization [7,86]. Owing to their capacity to simultaneously regulate multiple target genes, miRNAs contribute to the coordination of inflammatory responses, adaptive mechanisms, and structural alterations occurring in the myocardium during remodeling, thereby constituting an important element of the pathophysiology of cardiovascular diseases [87,88]. For instance, alterations in the expression of specific miRNAs may affect glucose uptake through regulation of glucose transporter expression, including GLUT1, which is essential for the activation of T lymphocytes and macrophages. At the same time, miRNAs modulate lipid metabolic pathways that determine both the production of inflammatory mediators and the maintenance of energy homeostasis in immune cells [89,90].

In humans, approximately 2500–2600 distinct miRNAs have been identified; however, only a limited subset appears to be of particular relevance to myocardial remodeling. One of the most extensively characterized miRNAs in the context of immunometabolism is miR-150, which plays a significant role in the development and function of lymphoid cells. Its expression correlates with the metabolic status of lymphocytes, indirectly affecting their proliferative capacity and immunological responsiveness [91]. By influencing lipid metabolism and the availability of energetic substrates, miR-122 may indirectly modulate immune cell function, particularly under conditions of chronic inflammation and metabolic dysregulation [90,92].

MiR-146 contributes to the maintenance of immunological homeostasis by restraining excessive activation of innate immune cells. Its activity is also associated with modulation of metabolic processes accompanying inflammatory activation, thereby facilitating metabolic adaptation of cells to conditions of immunological stress [93,94].

## 6. Therapeutic Perspectives

### 6.1. Modulation of Macrophage Metabolism

Accumulating evidence indicates that metabolic reprogramming of macrophages may represent an effective strategy to limit pathological inflammation during cardiac remodeling. Pro-inflammatory macrophages (M1 phenotype) are characterized by enhanced glycolytic activity, whereas reparative macrophages (M2 phenotype) rely predominantly on oxidative phosphorylation and fatty acid oxidation. These distinct metabolic programs are closely linked to functional polarization, and targeting these pathways may facilitate resolution of inflammation and support tissue repair following myocardial injury [95,96].

The clinical relevance of modulating inflammatory signaling in cardiovascular disease has been highlighted by the CANTOS trial, in which inhibition of interleukin-1β with canakinumab reduced recurrent cardiovascular events in patients with a history of myocardial infarction [97]. Greater reductions in inflammatory markers, particularly IL-6, were associated with more pronounced clinical benefits, underscoring the pivotal role of inflammatory pathways in cardiovascular pathology [98].

Additional support comes from studies of colchicine therapy. Both the COLCOT and LoDoCo2 trials reported significant reductions in major adverse cardiovascular events in patients receiving low-dose colchicine [99,100]. Although colchicine exerts multiple effects, its anti-inflammatory activity is believed to involve inhibition of microtubule-dependent inflammasome activation and suppression of IL-1β signaling. Collectively, these findings suggest that pharmacological modulation of macrophage-driven inflammation may help attenuate maladaptive cardiac remodeling.

### 6.2. Antifibrotic Therapeutic Targets—The TGF-β Pathway

Transforming growth factor-β (TGF-β) signaling plays a central role in fibroblast activation and differentiation into myofibroblasts, ultimately leading to excessive extracellular matrix deposition. Persistent activation of this pathway contributes to myocardial stiffening and structural remodeling, making it an attractive therapeutic target in fibrotic heart disease [82,101].

Several antifibrotic agents are currently under investigation in fibrotic disorders and may also have relevance in cardiovascular disease. For example, pirfenidone and nintedanib have been shown to inhibit fibroblast activation and extracellular matrix deposition in both experimental studies and clinical trials [102,103]. Although currently approved primarily for pulmonary fibrosis, their mechanisms suggest potential therapeutic utility in cardiac remodeling.

At the same time, the pleiotropic functions of TGF-β in tissue repair and immune regulation require careful consideration. Complete inhibition of this pathway may interfere with physiological healing processes, highlighting the need for selective approaches that modulate rather than fully suppress TGF-β signaling [104].

### 6.3. Mitochondria-Targeted Therapies

Mitochondrial dysfunction is a hallmark of the failing myocardium, leading to impaired ATP production, increased reactive oxygen species generation, and reduced metabolic flexibility of cardiomyocytes. These disturbances can amplify inflammatory signaling and accelerate maladaptive structural remodeling, making mitochondria a promising therapeutic target [105,106].

Sodium–glucose cotransporter-2 (SGLT2) inhibitors, such as empagliflozin and dapagliflozin, have emerged as an important class of cardioprotective agents. Clinical trials have demonstrated that these drugs reduce hospitalizations for heart failure and cardiovascular mortality, even in patients without diabetes [104,107].

Although initially developed as glucose-lowering medications, evidence indicates that their cardiovascular benefits extend beyond glycemic control. Proposed mechanisms include improved myocardial energy metabolism, enhanced fatty acid utilization, reduced oxidative stress, and increased mitochondrial efficiency. Additionally, these agents may indirectly influence inflammatory responses through modulation of immune cell metabolism, thereby contributing to attenuation of adverse cardiac remodeling [108].

### 6.4. MicroRNA-Based Therapies

MicroRNAs (miRNAs) represent a critical regulatory layer of gene expression and play a significant role in cardiac remodeling processes, including inflammation, fibrosis, and metabolic adaptation. Among them, miR-29 and miR-30 are notable for their ability to regulate extracellular matrix proteins and modulate fibroblast activity, suggesting potential antifibrotic effects [109,110].

Recent advances in RNA therapeutics have enabled strategies to modulate miRNA activity, including synthetic miRNA mimics and antisense oligonucleotides (antagomirs), aiming either to restore protective miRNA function or inhibit pathogenic miRNAs.

Accumulating evidence also indicates that miRNA dysregulation contributes to the pathogenesis of heart failure with preserved ejection fraction (HFpEF), a condition characterized by myocardial stiffening and interstitial fibrosis [111]. Beyond direct miRNA-targeted therapies, extracellular vesicles (EVs), including exosomes, are increasingly investigated as delivery systems for regulatory RNAs. Experimental studies suggest that EV-mediated transfer of cardioprotective miRNAs can attenuate inflammation and promote tissue repair after myocardial injury [112].

## 7. Conclusions

The issues presented in this work indicate that cardiac remodeling is a multidimensional process in which the close interplay between immune responses and metabolic reprogramming of cardiac cells plays a pivotal role. The immunometabolism of macrophages, fibroblasts, and cardiomyocytes represents a central mechanism regulating the balance between reparative processes and the development of pathological inflammation and fibrosis. Metabolic disturbances favor the persistence of pro-inflammatory and profibrotic phenotypes, ultimately leading to structural and functional cardiac dysfunction. At the same time, dynamic intercellular communication—mediated by cytokines, metabolites, microRNAs, and exosomes—integrates local inflammatory and energetic signals, thereby determining the course of remodeling. Understanding these interactions opens new therapeutic perspectives based on modulation of metabolic pathways, mitochondrial function, and epigenetic and post-transcriptional regulation. Immunometabolism thus emerges not only as a key component of the pathophysiology of cardiovascular diseases but also as a promising avenue for the development of future, more precise strategies for the treatment of heart failure and other cardiovascular diseases.

## Data Availability

No new data were created or analyzed in this study. Data sharing is not applicable to this article.
